# The Lifelong Impact of X-Linked Hypophosphatemia: Results From a Burden of Disease Survey

**DOI:** 10.1210/js.2018-00365

**Published:** 2019-05-07

**Authors:** Alison Skrinar, Melita Dvorak-Ewell, Ayla Evins, Carolyn Macica, Agnès Linglart, Erik A Imel, Christina Theodore-Oklota, Javier San Martin

**Affiliations:** 1 Ultragenyx Pharmaceutical Inc., Novato, California; 2 Frank H. Netter School of Medicine, Quinnipiac University, North Haven, Connecticut; 3 Hôpital Bicêtre, Le Kremlin-Bicêtre, Le Kremlin-Bicêtre, France; 4 Indiana University School of Medicine, Indianapolis, Indiana

**Keywords:** burden of disease, quality of life, XLH, X-linked hypophosphatemia

## Abstract

**Context:**

X-linked hypophosphatemia (XLH) is characterized by excess fibroblast growth factor 23 (FGF23), hypophosphatemia, skeletal abnormalities, and growth impairment. We aimed to understand the burden of disease of XLH across the lifespan.

**Methods:**

Responses were collected from adults with XLH and parents/caregivers of a child with XLH in an online survey, including multiple-choice and open-ended questions on demographics, disease manifestations, treatment history, assistive device use, and age-specific patient-reported outcomes (PROs).

**Results:**

Data were collected from 232 adults with XLH (mean age, 45.6 years; 76% female) and 90 parents/caregivers of a child with XLH (mean age, 9.1 years; 56% female). Mean age recalled for symptom onset was 3.2 years for adults and 1.3 years for children. When surveyed, nearly all children (99%) and 64% of adults were receiving oral phosphate, active vitamin D, or both. Prior participation in a trial investigating burosumab, a fully human monoclonal antibody against FGF23, was reported in 3% of children and 10% of adults; of these respondents, only one child reported current treatment with burosumab at the time of the survey. Both children and adults reported typical features of XLH, including abnormal gait (84% and 86%, respectively), bowing of the tibia/fibula (72% and 77%), and short stature (80% and 86%). Nearly all adults (97%) and children (80%) reported bone or joint pain/stiffness. Adults reported a history of fractures (n/N = 102/232; 44%), with a mean (SD) age at first fracture of 26 (16) years. Adults reported osteophytes (46%), enthesopathy (27%), and spinal stenosis (19%). Mean scores for PROs evaluating pain, stiffness, and physical function were worse than population norms. Analgesics were taken at least once a week by 67% of adults.

**Conclusions:**

Despite the common use of oral phosphate and active vitamin D established in the 1980s, children with XLH demonstrate a substantial disease burden, including pain and impaired physical functioning that persists, as demonstrated by similar responses reported in adults with XLH.

X-linked hypophosphatemia (XLH) is a rare, lifelong, often debilitating genetic disorder caused by loss-of-function mutations in the phosphate-regulating gene with homologies to endopeptidases on the X-chromosome (*PHEX*) [1]. XLH is estimated to occur in 1 in 20,000 live births [2–4]. Owing to *PHEX* deficiency, excess expression and secretion of fibroblast growth factor 23 (FGF23) causes hyperphosphaturia and chronic hypophosphatemia.

In children with XLH, the chronic hypophosphatemia causes osteomalacia and rickets, which are characterized by deficient bone mineralization, defective cartilage growth plate calcification, delayed endochondral ossification, and slowed growth velocity [5, 6]. In addition to rickets, children with XLH have lower extremity deformity, diminished growth, and pain. Dental issues, such as dental caries and abscesses, are also common in XLH due to defects in dentin and enamel [7]. Taken together, these factors may all contribute to a decreased health-related quality of life in children with XLH, but there is little research published to confirm this assumption.

In adults with XLH, the chronic hypophosphatemia leads to the progression of osteomalacia, and unresolved complications from childhood persist, such as lower extremity deformity and short stature. XLH in adults is also associated with increased risk of pseudofractures, osteoarthritis, enthesopathy, and a variety of musculoskeletal symptoms, including pain and stiffness that decrease mobility and impact daily function [1, 8–12]. One of the most common clinical consequences of osteomalacia is bone pain that can be generalized or localized [13, 14]. The severity of bone pain in adults with XLH has been shown to reflect the degree of osteomalacia evident by histomorphometry performed on biopsies of the transiliac crest of the hip [9]. In addition to bone pain, joint pain is common in adults with XLH and is caused by osteoarthritis that occurs as a long-term consequence of weight bearing on misaligned hips, knees, and ankles in adults.

Since the 1980s, children with XLH have managed their symptoms with multiple daily doses of oral phosphate often combined with active vitamin D (most commonly calcitriol or alfacalcidol). Although recent (2011 and 2014) guidelines are provided in the literature, age at initiation, dose, dose frequency, and adherence vary greatly among patients. The goal in the treatment of children is to improve bone mineralization, heal rickets, correct or minimize skeletal deformities, and maximize growth potential, and not specifically to normalize serum phosphorus levels. Skeletal outcomes vary with the use of oral phosphate and active vitamin D, with some children continuing to experience rickets, diminished height, lower extremity deformity, and corrective orthopedic surgeries [1, 6, 15, 16]. Although use of oral phosphate and active vitamin D is common practice for the management of children with XLH, no consensus exists regarding such application in adult patients with XLH due to limited efficacy related to slower rate of bone turnover and concerns over safety risks [1, 6]. As a result, use may be limited to those with chronic nontraumatic fractures, severe osteomalacia, and disabling skeletal pain, or to promote healing following orthopedic surgeries, such as joint replacement or fracture repair [1, 17, 18]. Long-term use of oral phosphate and active vitamin D can also pose serious risks in children and adults, such as nephrocalcinosis, hypercalciuria, and hyperparathyroidism, and therefore requires careful monitoring [4, 6].

Burosumab, a fully human monoclonal antibody against FGF23, was approved in 2018 for the treatment of XLH in the United States, European Union, and Canada (conditions of approval vary), >3 years after the surveys used in this study were created. In a phase 2 study in children with XLH, ages 5 to 12 years old, burosumab normalized fasting serum phosphorus levels, reduced rickets severity, and improved growth [19]. In another phase 2 study in children with XLH, ages 1 to 4 years old, burosumab also improved serum phosphorus levels, rickets, and leg bowing [20]. In a phase 3 study in adults with XLH, treatment with burosumab was associated with normalized serum phosphorus levels, increased markers of bone formation and remodeling, improved fracture healing, and reductions in stiffness [21].

Outside of the interventional trials with burosumab, most research has focused on the genetics, biochemistry, and skeletal presentation of XLH. Additional research is needed to fully understand the burden of the disease, particularly the impact on the lives of affected patients, in children and adults with XLH. Here, we report findings from an XLH Burden of Disease Study, a noninterventional, global, online survey of a large cohort of 232 children and adults with XLH. This survey was conducted in an effort to help assess the feasibility and appropriateness of the patient-reported outcomes (PROs) as endpoints for clinical trials investigating the efficacy and safety of burosumab for XLH.

## 1. Methods

### A. Study Population

All responders were required to be ≥18 years of age. The survey was designed to assess disease-related features in both children (via caregivers) and adults with XLH. The population surveyed included adults with a diagnosis of XLH or the parent, guardian, or caregiver of a child with a diagnosis of XLH. Respondents were asked to confirm diagnosis of XLH (for the child in the pediatric survey completed by the caregiver) and whether they had received a genetic confirmation of diagnosis (*PHEX* mutation); diagnosis was not verified with medical records. The survey instructed parents/caregivers to fill out one survey for each child when they cared for more than one child with XLH. Adults with XLH and parents/guardians/caregivers of children with XLH were recruited through the sponsor, The XLH Network Inc. (a disease-specific patient advocacy organization), and clinicians with a research interest in XLH or experience in the clinical management of individuals with XLH. More specifically, clinicians were identified by involvement in dose-finding phase 1/2 trials with burosumab, sponsored by Kyowa Hakko Kirin Co., Ltd., and by who had signed on for pediatric trials that were just beginning, sponsored by Ultragenyx Pharmaceutical Inc. Advertising for participation was provided to the members of The XLH Network Inc.; on the Web sites of the association pour les personnes atteintes de rachitisme vitamino-résistant hypophosphatemique and Ultragenyx Pharmaceutical Inc.; and via cards or brochures given to clinical providers. Cards given out at bone-specific conferences, such as the American Society of Bone and Mineral Research, had QR codes linked to the survey as well.

### B. Study Design and Methodology

The XLH Burden of Disease Survey was designed by a team of researchers employed by or affiliated with Ultragenyx Pharmaceutical Inc., in partnership with The XLH Network Inc. Questions and assessments in the survey (described in the PROs section below) were included based on recommendations from regulatory authorities, qualitative interviews with patients with XLH or caregivers [22], and literature investigating quality of life in diseases similar to XLH (*e.g.*, osteoarthritis) [23–26]. Data from this survey study and the open-label study UX023-CL203 (NCT2312687), which was initiated after the survey (March 2015), were used to validate these instruments (report submitted to the Food and Drug Administration available in an online repository [27]).

The XLH Burden of Disease Survey was accessed online and included an electronic consent form. Prospective participants were directed to a Web portal presenting an online consent form that required completion before accessing the survey. The survey was launched in English in June 2014, and was later translated into French, Spanish, Portuguese, and German. Findings from data collected through February 2016 are reported.

The adult version of the survey was to be completed by any adult with XLH ≥18 years of age. The parent/guardian/caregiver of a child with XLH <18 years of age completed the pediatric version of the survey on their behalf. Parents/guardians/caregivers were first asked to indicate their relationship to the affected child whose disease information was to be collected. Identifying information was not collected to ensure anonymity. The participant was required to complete the survey in one sitting. The questionnaire was designed to take ∼30 minutes.

Both versions of the survey, one for children (completed by a caregiver) and one for adults, included demographics, family history, diagnostic history, medical and surgical history, disease-specific clinical symptoms, treatments, medications, and assistive device use [27]. PROs were also used to assess pain, disability, and health-related quality of life (HRQoL). Data directly reflected respondents’ answers and were not verified against medical records. There was no direct interaction or querying of children, and thus they did not provide formal assent.

The protocol, consent form, and participant materials for the XLH Burden of Disease Survey were reviewed and approved by the New England Institutional Review Board. Each patient, his or her parents, or a legal representative provided informed consent for publication of data. Requests for individual deidentified participant data and the protocol from this study will be available for at least 12 months after publication to researchers providing a methodologically sound proposal that is in accordance with the Ultragenyx’s data-sharing policy. To gain access, data requestors will need to sign a data access and use agreement. Data will be shared via a secured portal.

### C. PROs

The adult version of the survey included the following PROs: the Western Ontario and McMaster Universities Osteoarthritis Index (WOMAC^®^), the Brief Pain Inventory (BPI) (short form), and the 36-Item Short Form Health Survey version 2 (SF-36v2) [28–30]. The WOMAC assessed pain, stiffness, and physical function in adults. The BPI assessed pain severity and pain interference with daily life. SF-36v2 assessed general HRQoL in adults.

The pediatric version of the survey included the Pediatric Outcomes Data Collection Instrument (PODCI) developed by the Pediatric Orthopedic Society of North America and the 10-Item Short Form Health Survey (SF-10) [31, 32]. The PODCI was used to assess disability concepts in children. The SF-10 was used to assess general HRQoL in children.

Minimally important differences, defined as the smallest change in an outcome that a patient would identify as important or clinically meaningful, from PRO instrument manuals and the literature were used to interpret PRO results [28–32].

### D. Statistical Analysis

Demographics and disease characteristics for adult and pediatric populations were separately examined using frequency distributions and summary measures. Height-for-age *z* scores and percentiles that standardize height for age and sex were calculated using clinical growth chart normative data from the Centers for Disease Control/National Center for Health Statistics [33]. PRO scores are reported as the estimated sample means alongside US population norms [28–32].

## 2. Results

### A. Study Population

Respondents were comprised of 90 caregivers (representing 90 children with XLH) and 232 adults with XLH (Table 1). Although survey responses were received from >30 countries, most responders were from the United States [154 (67%) adults; 42 (47%) children]. The range of ages for children was 1 to 18 years, and the range for adults was 18 to 74 years, suggesting representation across the lifespan in this cross-sectional, convenience sample. Despite instructions on the age limitation of the pediatric survey, both the adult and pediatric survey included one 18-year-old patient; the responses for the 18-year-old in the pediatric survey were included in the pediatric analysis and not combined with the adult analysis, as the content of the two surveys differed.

**Table 1.** Demographics and Characteristics of the Study Population

Demographic Characteristics	Children*^a^* (n = 90)	Adults (n = 232)
Age, y, mean (SD)	9.1 (3.9)	45.6 (12.9)
Female, n (%)	50 (55.6)	177 (76.3)
Age at symptom onset, y, mean (SD)	1.3 (1.9)	3.2 (7.2)
Age at diagnosis of XLH, y, mean (SD)	2 (2.2)	9.3 (13.5)
Self-reported *PHEX* mutation, n (%)	53 (58.9)	90 (39.0)
Current use of oral phosphate and active vitamin D, n (%)	89 (98.9)	110 (47.4)
Current use of oral phosphate, n (%)	89 (98.9)	114 (49.1)
Current use of active vitamin D, n (%)	89 (98.9)	149 (64.2)
Previous participation in a clinical trial with burosumab, n (%)	3 (3.3)	23 (9.9)
Current use of burosumab, n (%)	1 (0.1)	0 (0.0)

^a^ Note that a caregiver filled out the pediatric survey for an 18.9-y-old individual with XLH and the responses are included in the pediatric dataset.

### B. Current Management

At the time of survey completion, 99% (89/90) of children and 49% (114/232) of adults were being treated with oral phosphate; 99% (89/90) of children and 64% (149/232) of adults were being treated with active vitamin D metabolites; 99% (89/90) of children and 47% (110/232) of adults were receiving both oral phosphate and active vitamin D (Table 1). At the time of survey completion, 3% (3/90) of children and 10% (23/232) of adults had participated in a clinical trial with burosumab; 1 out of 3 of these children and none of these 23 adults reported currently receiving burosumab. Given the dates of the survey (June 2014 to February 2016), the adults who reported receiving burosumab likely participated in a phase 1 or 2 dose-finding study (NCT01340482, NCT01571596) sponsored by Kyowa Hakko Kirin Co., Ltd.

Data regarding management of disease was only collected regarding current use and previous participation in a clinical trial with burosumab; information regarding age of initiation with treatment, dose of treatment, adherence to treatment, and duration of treatment were not collected. Because these details were not collected and the survey focused on questions that occurred at any point in the patient’s medical history, and not specifically at the time of treatment (with oral phosphate and active vitamin D or burosumab), no subanalyses comparing outcomes by treatment regimen were conducted.

### C. Growth and Stature

The box was checked for “short stature” for 80% (72/90) of respondents for the pediatric survey and 86% (200/232) of respondents for the adult survey. Impaired growth was evident in early childhood and persisted through adolescence and adulthood (Table 2). Bowing of the lower extremities, which exacerbates the diminished height caused by delayed growth of the long bones, was common, with bowing of the tibia and/or fibula reported for 72% (65/90) of children and 77% (178/232) of adults, and bowing of the femur was reported for 63% (57/90) of children and 66% (152/232) of adults. Genu valgum (“knock knees”) was reported for 32% (29/90) of children and 27% (63/232) of adults.

**Table 2.** Height in Patients With XLH

Age Group	n	Standing Height (cm)	Percentile	Height-for-Age *z* Score
Females*^a^*				
2–4 y old	4	90.6 (3.6)	8.6 (3.4)	−1.4 (0.25)
5–12 y old	32	120.9 (13.3)	13.0 (23.9)	−1.7 (1.6)
13–17 y old	11	150.7 (10.8)	21.4 (26.6)	−1.5 (1.7)
18 y or older	164	148.2 (8.7)	6.5 (12.5)	−2.4 (1.3)
Males*^b^*				
2–4 y old	9	88.4 (10.3)	12.3 (31.6)	−2.8 (2.3)
5–12 y old	25	122.9 (19.5)	16.7 (22.3)	−1.9 (2.0)
13–17 y old	5	153.8 (9.1)	4.0 (2.5)	−1.9 (0.4)
18 y or older	54	162.5 (9.6)	10.5 (18.6)	−2.0 (1.3)

Results are shown as mean (SD).

^a^ Responses for 3 children and 13 adults were excluded due to aberrant responses that indicated a misunderstanding of the question.

^b^ Responses for one child and one adult were excluded because the age falls outside the likely range of subgroup analysis and an aberrant response indicated a misunderstanding of the question, respectively.

### D. Surgical History

Surgical interventions were reported for 47% of children and 94% of adults (Table 3). More than half (61%) of adults and 17% of children reported a history of osteotomy, an invasive procedure to correct deformity in the lower extremities typically performed after the cessation of growth. Stapling of the growth plate, an increasingly common, less invasive procedure aimed at correcting angular defects during growth was reported for 19% of children and 6% of adults. Adults reported fracture repair (discussed in greater detail in following section) and joint replacements, likely associated with progression of osteomalacia and osteoarthritis. Cartilage and fracture repairs, which may arise from the burden of weight bearing on misaligned joints, were reported in adults noting “other” surgeries.

**Table 3.** XLH Surgical History

Surgical Procedure	Children, n (%)*^a^*	Adults, n (%)*^a^*
Osteotomy	15 (16.7)	142 (61.2)
Stapling of growth plates	12 (13.3)	14 (6.0)
Skull surgery (craniotomy/craniectomy)	3 (3.3)	6 (2.6)
Knee replacement	N/A	21 (9.1)
Hip replacement	N/A	16 (6.9)
Cartilage repair*^b^*	N/A	18 (7.8)
Fracture repair*^b^*	N/A	13 (5.6)

Abbreviation: N/A, not assessed.

^a^ Percentage is calculated from the total pediatric population (n = 90) for pediatric surgeries and from the total adult population (n = 232) for adult surgeries.

^b^ Cartilage and fracture repair were calculated from free response answers given in response to “Other.”

### E. Skeletal Health

Forty-four percent (102/232) of adult responders indicated a history of fracture (not distinguishing between fracture and pseudofracture), particularly in the lower extremities, a common complication in adults caused by progressive osteomalacia and the impact of prolonged weight bearing on poorly mineralized bones and misaligned joints. For adults reporting a history of fracture, the mean (SD) age at first fracture was 26.4 (15.7) years. Although the average age of adults reporting a history of fracture was 48.4 years, 42% (43/102) of these responders reported at least three fractures. Fractures were most common in the lower extremities (Table 4). Fracture history was not assessed in the pediatric survey, as nontraumatic fractures are not common in children.

**Table 4.** Fracture History in Adults With XLH

History of Fracture, n/N (%)*^a^*		Number of Subjects With Fractures, n*^a^*					Age at First Fracture (y), Mean (SD)*^a^*
		1	2	3	4	5+	
All Locations	102/232 (44)	**—**	**—**	**—**	**—**	**—**	**—**
Femur (thigh)	43/232 (18.5)	21	9	6	0	6	29.3 (16.0)
Feet	32/232 (13.8)	20	4	3	1	4	36.9 (13.2)
Tibia/fibula (shin)	30/232 (12.9)	16	7	1	1	5	19.8 (12.7)
Hip	16/232 (6.9)	8	4	3	0	1	37.9 (16.3)
Hand/wrist	14/232 (6.0)	9	4	0	0	1	25.8 (18.2)
Forearm	10/232 (4.3)	1	2	3	3	1	27.8 (18.7)
Fingers	8/232 (3.4)	4	1	1	0	2	23.0 (8.1)
Back	6/232 (2.6)	3	1	0	0	2	39.6 (18.7)

Three participants indicated a history of fractures, but did not provide details on the location, frequency, or age of fracture occurrence.

^a^ Mean (SD) age of first fracture is calculated from only participants that indicated a history of fracture at that location. Sixteen subjects who indicated a history of fractures did not provide their age at the time of the fracture.

In the adult survey, 46%, 27%, and 19% of respondents reported osteophytes (bone spurs), enthesopathy (calcifications of tendons and ligaments), and spinal stenosis (narrowing of the spinal canal), respectively; the frequency of reporting increased with age (Table 5).

**Table 5.** Osteophytes, Enthesopathy, and Spinal Stenosis in Adults With XLH

Age (y)	n	Osteophytes, n (%)*^a^*	Enthesopathy, n (%)*^a^*	Spinal Stenosis, n (%)*^a^*
**All**	232	106 (46)	63 (27)	44 (19)
18–20	5	1 (20.0)	0 (0.0)	0 (0.0)
21–30	29	6 (20.7)	2 (6.9)	1 (3.5)
31–40	50	22 (44.0)	13 (26.0)	6 (12.0)
41–50	65	32 (49.2)	17 (26.2)	13 (20.0)
51–60	52	29 (55.8)	18 (34.6)	11 (21.2)
60+	31	16 (51.6)	13 (41.9)	13 (41.9)

^a^ Percentage per age group is based on the total number of participants within that age group.

### F. Dental Complications

Dental abscesses were reported in 51% (46/90) of children and 82% (189/232) of adults. Excessive cavities were reported in 24% (22/90) of children and 52% (121/232) of adults. A history of root canal surgery was reported for 17% (15/90) of children and 72% (166/232) of adults. Twenty-two of the adults surveyed reported failure of dental implants.

### G. Other Clinical Complications

Other complications of XLH reported included tinnitus [9% (8/90) of children and 46% (106/232) of adults], hearing loss [8% (7/90) of children and 34% (78/232) of adults], craniotomy or craniectomy [3% (3/90) of children and 6% (14/232) of adults], and Chiari malformations [8% in both children (7/90) and adults (18/232)]. These conditions have been reported previously in the literature, although less is known about the mechanism [34, 35].

Complications previously associated with the use of oral phosphate and active vitamin D [1, 4, 6, 15] were reported in this cohort of adults and children, including nephrocalcinosis [21% (49/232) of adults and 32% (29/90) of children], nephrolithiasis (kidney stones) [14% (32/232) of adults and 2% (2/90) of children], and impaired renal function [8% (19/232) of adults and 1 child].

Hyperparathyroidism was reported in 29% (68/232) of adults and 18% (16/90) of children. Use of calcimimetics was reported by 16% (11/68) of the adults with hyperparathyroidism, whereas 26% (18/68) of those with hyperparathyroidism underwent parathyroidectomy.

### H. Pain, Stiffness, and Functional Limitations

Pain was highly prevalent among adults and children (Table 6). Nearly all adults (97%) and children (80%) reported having experienced bone or joint pain in the year prior to taking the survey. Muscle pain was also reported in 63% of adults and 60% of children.

**Table 6.** Bone and Joint Pain in Children and Adults With XLH

Location	Bone Pain, n (%)		Joint Pain, n (%)	
	Adults	Children	Adults	Children
Back	69 (29.7)	8 (8.9)	120 (51.7)	11 (12.2)
Hips	92 (39.7)	10 (11.1)	137 (59.1)	13 (14.4)
Upper leg (thigh)	74 (31.9)	28 (31.1)	**—**	**—**
Knee	106 (45.7)	30 (33.3)	177 (76.3)	51 (56.7)
Lower leg (shin)	78 (33.6)	26 (28.9)	**—**	**—**
Ankle	74 (31.9)	19 (21.1)	119 (51.3)	31 (34.4)
Feet	81 (34.9)	17 (18.9)	94 (40.5)	15 (16.7)
Toes	27 (11.6)	3 (3.3)	47 (20.3)	5 (5.6)

**Figure 1.**
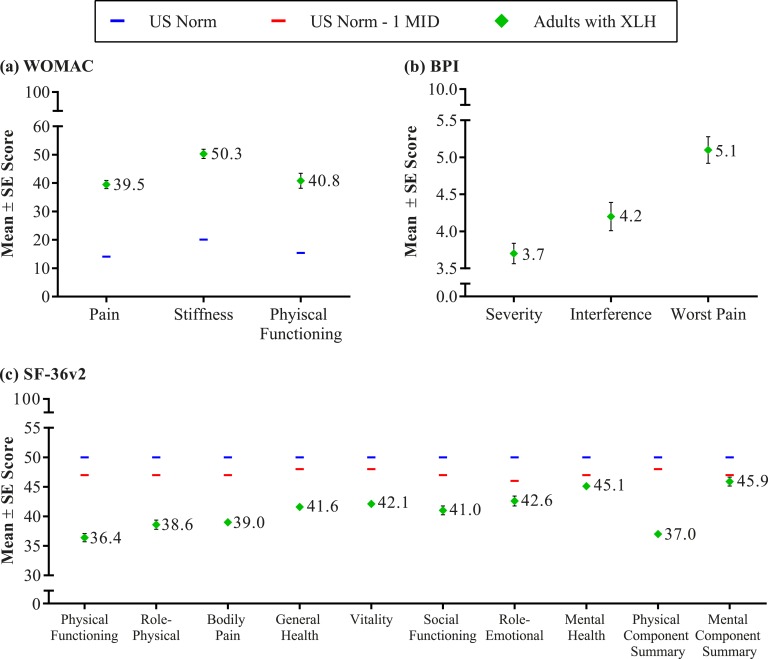
Functioning and HRQoL in adults with XLH. (a) WOMAC results by domain; higher scores indicate worse pain, stiffness, and functioning. (b) BPI results by domain; mild scores range from 0 to 3, moderate scores range from 3 to 6, and severe scores range from 6 to 10. (c) SF-36v2 results by domain. Some SE values are too small to be visible on the graph (*i.e.*, <1.0). MID, minimally important difference.

In 67% (156/232) of adult responders, pain was severe enough to warrant the use of medication at least once per week; this includes 69% (159/232) reporting over-the-counter pain medication use (nonsteroidal anti-inflammatory drugs, naproxen sodium, and acetaminophen) and 21% (48/232) reporting prescription pain medication use (oxycodone, oxycodone and acetaminophen, acetaminophen with codeine, hydrocodone bitartrate, cortisone, and hydrocodone bitartrate and acetaminophen). Despite the use of these medications, mean BPI pain severity, BPI worst pain (pain at its worst in the past 24 hours), and pain interference scores were 3.7, 5.1, and 4.2, respectively, indicating pain of moderate severity that interferes with daily function (Fig. 1). Pain ratings were higher for adults who reported a history of fracture with BPI pain severity, pain at its worst, and pain interference scores of 4.0, 5.6, and 4.9, respectively. Pain medication use is not common in children and was not assessed in the pediatric survey.

Joint stiffness or restricted range of motion was reported for 38% (34/90) of children and 91% (210/232) of adults. Osteoarthritis, which likely contributed to the occurrence of joint pain and stiffness, was reported by 54% (126/232) of adults. The mean WOMAC stiffness domain score reported by adult responders was 50.3, notably higher than the population normative score of 20.1 (Fig. 1) [36].

**Figure 2.**
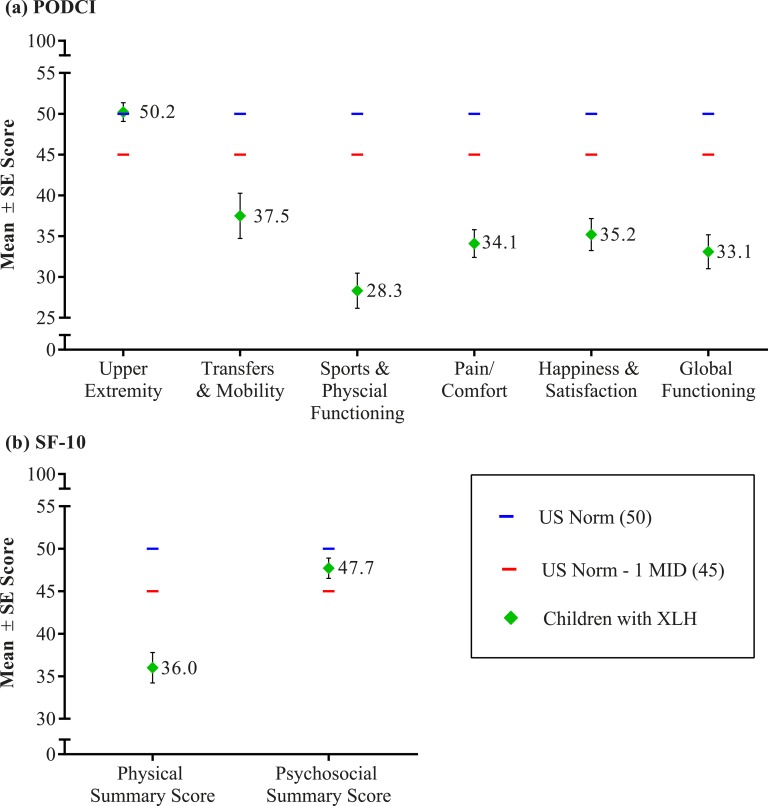
Functioning and HRQoL in children with XLH. (a) PODCI results by domain. (b) SF-10 results by summary score. MID, minimally important difference.

Most survey responders reported a history of clinical symptoms and conditions that can impact mobility, including delayed walking [44% (40/90) of children], an unusual gait or way of walking/running [84% (76/90) of children, 86% (199/232) of adults], muscle weakness [30% (27/90) of children, 60% (140/232) of adults], and walking device use [10% (9/90) of children, 31% (72/232) of adults]. Parents or caregivers of children reported PODCI scores for the transfers/basic mobility, sports/physical function, and pain/comfort domains between 1 and 2 SDs below the US general population norm (Fig. 2). Only the PODCI upper extremity function domain mean score was within normal limits. The global function score, a composite score based on scores from these four domains, was also nearly 2 SDs below the US general population norm. The impairment detected in the PODCI scores reflects the impact of the skeletal disease, particularly the lower extremity deformity, on the daily function of affected children.

Similar to the children, the physical limitations reported by adults cause substantial disability as evidenced by a WOMAC physical function domain score of 40.8, which is notably higher than the population normative score of 15.4 (Fig. 1).

### I. HRQoL

Generic HRQoL measures also reflect the impact of XLH on the lives of affected children and adults and allow for a comparison with other diseases that cause pain and impaired mobility. The mean (SD) SF-10 physical health summary score in children was 36.0 (15.8), nearly 1.5 SDs below the US general population norm of 50 [10]. The mean (SD) SF-10 psychosocial summary score of 47.7 (10.5) was in the normal range [mean (SD) of 50 (10)]. Diminished HRQoL was also noted in adults with a mean (SD) SF-36v2 physical component summary (PCS) score of 37.0 (10.2), >1 SD below the norm of 50 (Fig. 1). Physical functioning, role-physical, bodily pain, and general health subscale scores, which contribute most heavily to the calculation of the PCS score, also showed impairment. Vitality, social function, role-emotional, and mental health subscale scores, which contribute most heavily to the calculation of the MCS score, were within 1 SD of the US general population norm, indicating no clinically meaningful impairment in mental health status. The SF-36v2 PCS and subscale scores are consistent with other conditions known to impact physical functioning and HRQoL, such as back pain, osteoarthritis, and axial spondyloarthritis [8, 37, 38].

In response to the question on the adult survey, “If you had to spend the rest of your life with this condition as it is right now, how would you feel about it?”, respondents answered 30% very dissatisfied, 30% somewhat dissatisfied, 21% neutral, 14% somewhat satisfied, and 5% very satisfied.

## 3. Discussion

We employed the use of an online survey to better understand the burden of disease, gain perspective from a global XLH population and their caregivers, and inform study design while in early clinical development of trials with burosumab. Despite the use of oral phosphate and active vitamin D as standard clinical practice for the management of affected children since the 1980s, most of the survey respondents reported short stature, gait abnormalities, bowing in the lower extremities, dental abscesses, pain, impaired mobility, and diminished physical health status. Adults reported comorbidities associated with unresolved childhood disease, including short stature, lower extremity deformity, and impaired mobility. Additionally, adults reported complications consistent with progressive osteomalacia, including fractures, orthopedic surgery, and pain, as well as osteoarthritis, impaired mobility, and physical impairment. These findings emphasize the lifelong impact of XLH.

In addition to confirming the high prevalence of bowing, corrective surgeries, and short stature seen in the literature, survey responses for children with XLH characterized impairments that are not as well documented, including issues with mobility, gross motor function, and pain [39–43]. Complications of XLH can impact mobility in children and adults and ultimately reduce the ability to perform basic activities of daily living, including walking, dressing, and personal hygiene [9, 10, 44, 45]. Adding to studies that advocate for early diagnosis and intervention, low height for age *z* scores suggest that height decrements begin early in life [16, 46]. The most notable pediatric survey response was the low sports and physical function score reported on the PODCI, a robust measure of pediatric health status that correlates strongly with overall HRQoL [47, 48]. A child’s ability to engage in physical activities with peers is an important aspect of development; thus, the low scores on this domain that suggest impaired gross motor function could have a substantial psychosocial impact on children with XLH.

With the lack of recent literature on XLH in adults, survey results provided a much needed insight into the impact of XLH in adulthood [9–11, 45]. Adults reported osteoarthritis, osteomalacia, enthesopathy, and recurrent fractures, particularly in the lower extremities. Other studies have reported a lower incidence of fractures, but higher incidence of pseudofractures in XLH [10, 11, 44]. The survey could not distinguish between pseudofractures (typical of osteomalacia) and regular fractures, and patients might not be able to reliably distinguish between them. Pseudofractures among adults in this cohort may likely be caused by prolonged weight bearing on misaligned joints and poorly mineralized bones. Consistent with the high prevalence of pseudofractures, adults reported substantial bone pain. Collectively, the adult findings provide context for the noted impairments in pain, stiffness, physical functioning, and HRQoL as measured by the BPI, WOMAC, and SF-36v2.

More adults than children reported a history of osteotomy (61% vs 17%), but the rate of this procedure will likely increase as growth continues given the mean age of the pediatric cohort (9.1 years). Such findings also suggest that unresolved childhood disease may lead to surgical intervention. The high rate of surgical procedures in the adult respondents is supported by a study conducted by Berndt *et al.* [44] that reported 13 of 23 patients with XLH had a total of 101 orthopedic operations.

Adults reported recalling their first symptoms and age of diagnosis later than those of the children. This discrepancy may be due to a lack of information and awareness of XLH in the years prior to 1980 or to misremembering in the adult population because more time has elapsed since initial symptoms would have appeared.

Although oral phosphate and active vitamin D became the standard of care for children with XLH in the 1980s, debilitating disease was still prevalent among survey respondents, highlighting a substantial unmet medical need for better XLH treatment options. The success of oral phosphate and active vitamin D in children is limited by the inconvenience of multiple daily dosing, required surveillance, variability of skeletal response, and side effects [6]. Variability of response may also be due to a lack of standardization among use of oral phosphate and vitamin D (age at initiation, dose, and dose frequency vary greatly among patients) or may be because supplementation therapy provides transient increases in serum phosphorus and does not directly address the underlying pathophysiology of XLH [1, 6]. Findings from this survey were consistent with previous research using qualitative interviews that documented the side effects and the impact of the regimen of oral phosphate and active vitamin D on various aspects of HRQoL in children, including sleep, playdates, and school. Complications previously associated with oral phosphate and active vitamin D were reflected in this survey, including nephrocalcinosis and hyperparathyroidism [1, 4, 6].

At the time this survey was available, the efficacy of burosumab was not established, only a small number of respondents (8%) reported prior participation in a clinical trial with burosumab, and only one respondent reported current treatment with burosumab during the survey. We also cannot confirm the duration of exposure to burosumab for these respondents, although it is likely limited to that of a dose-finding, phase 1/2 study (NCT01340482, NCT01571596) sponsored by Kyowa Hakko Kirin Co., Ltd because of the timing for which this survey occurred. Ultragenyx had not initiated clinical trials in adults with XLH, and results from pediatric studies sponsored by Ultragenyx were not yet available. For these reasons and because the survey focused on questions that occurred at any point in the patient’s medical history, and not specifically at the time of treatment, no conclusions can be made regarding burosumab’s impact on burden of disease. Rather, the efficacy of burosumab is better addressed in controlled clinical trials [19–21].

Although this study is the largest survey of patients with XLH conducted to date, there are limitations associated with the survey methodology. Collaborations with physicians and patient advocacy groups, such as The XLH Network Inc., were used to encourage participation. Individuals associated with an advocacy group and/or receiving regular medical care may not reflect the larger population of individuals with XLH; certain types of patients, perhaps those with greater disease severity, may be more likely to participate in research that may contribute to a greater understanding of disease, such as this online survey. Additionally, information relied on responder recall and could not be verified by medical records, as patient identifiers were not collected. Similarly, compliance with conventional therapy, which can substantially impact clinical outcomes, could not be verified. Our questionnaires may have not collected data on all possible variables that impact burden of disease or adherence to treatment. Still, our results support and expand on findings reported in the literature describing significant disease burden despite management with oral phosphate and active vitamin D during childhood. Although these limitations are common to survey methodology, patient-reported data are also the most reliable source for insight into daily function, HRQoL, and symptoms that are only known to the patient, such as pain, stiffness, and impaired mobility [49]. Lastly, this study provided a much-needed insight into the burden of disease of XLH from childhood into adulthood.

## 4. Conclusions

The XLH Burden of Disease Survey Study expands our understanding XLH from the patient perspective. Results suggest that both children and adults with XLH are living with a chronic, progressive, and debilitating disease that causes pain and impacts mobility and health-related quality of life. Unresolved symptoms and conditions experienced in childhood lead to substantial complications in adulthood and additional complications emerge as the disease progresses.

## Abbreviations:

BPI: Brief Pain Inventory
FGF23: fibroblast growth factor 23
HRQoL: health-related quality of life
PCS: physical component summary
PHEX: phosphate-regulating gene with homologies to endopeptidases on the X-chromosome
PODCI: Pediatrics Outcomes Data Collection Instrument
PRO: patient-reported outcome
SF-10: 10-Item Short Form Health Survey
SF-36v2: Short-Form Health Survey 36 version 2
WOMAC: Western Ontario and McMaster Universities Osteoarthritis Index
XLH: X-linked hypophosphatemia
